# Acceptability and Feasibility of the Telehealth Bariatric Behavioral Intervention to Increase Physical Activity: Protocol for a Single-Case Experimental Study

**DOI:** 10.2196/39633

**Published:** 2022-09-29

**Authors:** Aurélie Baillot, Maxime St-Pierre, Josyanne Lapointe, Paquito Bernard, Dale Bond, Ahmed Jérôme Romain, Pierre Y Garneau, Laurent Biertho, André Tchernof, Patricia Blackburn, Marie-France Langlois, Jennifer Brunet

**Affiliations:** 1 Nursing Department Université du Québec en Outaouais Gatineau, QC Canada; 2 Institut du savoir de l’hôpital Montfort-recherche Ottawa, ON Canada; 3 Centre de Recherche en Médecine Psychosociale Centre Intégré de Santé et Services Sociaux de l’Outaouais Gatineau, QC Canada; 4 Basic Science Department Université du Québec à Chicoutimi Chicoutimi, QC Canada; 5 Department of Physical Activity Sciences Université du Québec à Montréal Montréal, QC Canada; 6 Montreal Mental Health University Institute Research Centre Montreal, QC Canada; 7 Department of Surgery Hartford Hospital/HealthCare Hartford, CT United States; 8 School of Kinesiology and Physical Activity Sciences Faculty of Medicine Université de Montréal Montréal, QC Canada; 9 Department of Surgery Université de Montréal Montréal, QC Canada; 10 Institut universitaire de cardiologie et de pneumologie de Québec Québec, QC Canada; 11 Division of Kinesiology Department of Health Sciences Université du Québec à Chicoutimi Chicoutimi, QC Canada; 12 CHUS Research Center and Division of Endocrinology Department of Medicine, Faculty of Medicine and Health Sciences Université de Sherbrooke Sherbrooke, QC Canada; 13 Faculty of Health Sciences School of Human Kinetics University of Ottawa Ottawa, ON Canada; 14 Cancer Therapeutic Program Ottawa Hospital Research Institute Ottawa, ON Canada

**Keywords:** behavior change intervention, bariatric surgery, physical activity, telehealth, single-case experimental design, self-determination, self-efficacy, behavior change techniques, mobile phone

## Abstract

**Background:**

Regular physical activity (PA) is recommended to optimize weight and health outcomes in patients who have undergone metabolic and bariatric surgery (MBS). However, >70% of patients have low PA levels before MBS that persist after MBS. Although behavioral interventions delivered face-to-face have shown promise for increasing PA among patients who have undergone MBS, many may experience barriers, preventing enrollment into and adherence to such interventions. Delivering PA behavior change interventions via telehealth to patients who have undergone MBS may be an effective strategy to increase accessibility and reach, as well as adherence.

**Objective:**

This paper reports the protocol for a study that aims to assess the feasibility and acceptability of the protocol or methods and the Telehealth Bariatric Behavioral Intervention (TELE-BariACTIV). The intervention is designed to increase moderate-to-vigorous intensity PA (MVPA) in patients awaiting bariatric surgery and is guided by a multitheory approach and a patient perspective. Another objective is to estimate the effect of the TELE-BariACTIV intervention on presurgical MVPA to determine the appropriate sample size for a multicenter trial.

**Methods:**

This study is a multicenter trial using a repeated (ABAB’A) single-case experimental design. The A phases are observational phases without intervention (A1=pre-MBS phase; A2=length personalized according to the MBS date; A3=7 months post-MBS phase). The B phases are interventional phases with PA counseling (B1=6 weekly pre-MBS sessions; B2=3 monthly sessions starting 3 months after MBS). The target sample size is set to 12. Participants are inactive adults awaiting sleeve gastrectomy who have access to a computer with internet and an interface with a camera. The participants are randomly allocated to a 1- or 2-week baseline period (A1). Protocol and intervention feasibility and acceptability (primary outcomes) will be assessed by recording missing data, refusal, recruitment, retention, attendance, and attrition rates, as well as via web-based acceptability questionnaires and semistructured interviews. Data collected via accelerometry (7-14 days) on 8 occasions and via questionnaires on 10 occasions will be analyzed to estimate the effect of the intervention on MVPA. Generalization measures assessing the quality of life, anxiety and depressive symptoms, and theory-based constructs (ie, motivational regulations for PA, self-efficacy to overcome barriers to PA, basic psychological needs satisfaction and frustration, PA enjoyment, and social support for PA; secondary outcomes for a future large-scale trial) will be completed via web-based questionnaires on 6-10 occasions. The institutional review board provided ethics approval for the study in June 2021.

**Results:**

Recruitment began in September 2021, and all the participants were enrolled (n=12). Data collection is expected to end in fall 2023, depending on the MBS date of the recruited participants.

**Conclusions:**

The TELE-BariACTIV intervention has the potential for implementation across multiple settings owing to its collaborative construction that can be offered remotely.

**International Registered Report Identifier (IRRID):**

DERR1-10.2196/39633

## Introduction

### Background

Severe obesity (class II and III; BMI ≥35 kg/m^2^) affects approximately 9.5% of Canadians [1]. Metabolic and bariatric surgery (MBS) is generally effective in inducing significant and lasting weight loss [2] while reducing the costs related to severe obesity and associated complications [3,4]. MBS offers significant health benefits and improves the quality of life [4,5]. The number of procedures performed worldwide has increased during the last decade in particular, reaching almost 700,000 surgeries performed in 2018 [6]. However, approximately half of the patients who have undergone MBS regain at least 10% and 20% of their maximum weight loss 1 year and 5 years after reaching their nadir weight, respectively [7].

In the context of MBS, physical activity (PA) is recommended given its multiple benefits [8,9]. Studies have shown that pre- and post-MBS PA can improve physical fitness, cardiometabolic health, quality of life, and depressive symptoms [10-17]. However, 70% to 95% of adults are inactive before MBS and remain so after MBS [18,19]. Thus, there is a need to develop interventions with the aim of increasing pre- and post-MBS PA. Introducing PA behavior change interventions before MBS with additional support offered after MBS seems to be an ideal approach to optimize MBS benefits and provide transitional support toward post-MBS lifestyle [20]. The most compelling evidence to support this assertion comes from studies that have shown that PA interventions delivered before MBS can increase PA until 1 year after MBS [21,22]. Furthermore, pre-MBS PA can improve physical fitness, which may help reduce perioperative complications and length of hospital stay [23]. Finally, participants’ motivation and readiness for PA increased close to their surgical date [24].

Supervised exercise within the context of clinical- and research-based interventions, although effective in conferring health benefits, does not increase long-term PA in adults undergoing MBS [17,25,26], likely because this type of intervention almost never includes behavioral change methods (ie, no target of PA determinants such as self-determination and self-efficacy that may help with maintenance of PA behavior). Interventions that apply methods specifically targeting PA determinants are needed to equip adults with the knowledge, skills, and confidence to engage in regular PA on their own, as resources are not infinite. Therefore, PA behavior change interventions should be considered, which can be effective in increasing PA in adults with obesity [27]. In adults undergoing MBS, Bond et al [22,28,29] have shown that BariActiv, a 6-week intervention delivered before MBS, which included weekly one-on-one meetings and focused on behavior change techniques (BCTs), increased moderate-to-vigorous intensity PA (MVPA) and step counts after the intervention, and these changes were sustained 6 months after MBS. Thus, PA behavior change interventions seem promising to increase MVPA in the midterm and, in turn, limit weight regain to maintain health improvements. However, additional studies investigating the effects of behavior change interventions on MVPA are required owing to the heterogeneity and small number of studies available and the divergent results across studies with adults undergoing MBS [30].

### Successful Behavior Change Interventions Targeting PA

#### Overview

Successful behavior change interventions are based on evidence and a good understanding of the targeted behavior and its correlates, context, and population [31,32]. Such interventions also require input from stakeholders, especially those who will receive it, which can be obtained via various methods. For example, to inform theory- and evidence-based decisions about the design and delivery of PA behavior change interventions, a web-based survey and focus groups were conducted with adults undergoing MBS living in Quebec, Canada [33]. The results highlight 4 fundamental points to consider when developing a behavior change intervention to promote PA among adults undergoing MBS.

#### Needs Assessment and Patient Perspectives

First, the main PA motives before and after MBS include weight control, social support, pleasure, well-being, and physical fitness [34-40]. Therefore, PA interventions should identify strategies addressing these motives to meet patients’ expectations and keep track of these for efficacy evaluations. Second, motivating adults to engage in PA, either before or after MBS, is challenging because of several barriers [41-44], including physical and psychological issues, such as pain, fatigue, weight, lack of motivation, and body dissatisfaction [34-40]. Other important PA barriers reported include perceived lack of time and resources, bad weather, and lack of social support [34-40], which can persist after MBS despite weight loss and health improvement [45]. In addition, new PA barriers may emerge after MBS caused by excess skin [46,47]. Accordingly, adults are likely to need support to overcome barriers to PA both before and after MBS. Ensuring that a well-trained PA counselor who can help intended users elucidate barriers and identify means to create an environment that makes PA easier and reduces barriers to action is important. Third, adults reported a preference for PA interventions starting 3 to 6 months before MBS and being offered continued support until 1 year after MBS [33], suggesting that initiating a PA behavior change intervention before MBS, with additional post-MBS follow-ups, may be most acceptable. Fourth, interventions offering face-to-face meetings with health professionals are preferable [33]. Accordingly, integration of supervision via regular meetings should thus be considered as a key element to enhance participants’ adherence to the intervention and, in turn, their PA [48,49].

#### Multitheory Approach

Beyond the aforementioned considerations, it is necessary to draw on behavior change theories to target specific factors that influence PA behavior when developing PA behavior change interventions. Existing theories such as self-determination theory (SDT) [50] and social cognitive theory (SCT) [51] are 2 theories previously used in PA behavior change interventions [52], as they are especially relevant in identifying determinants of PA. SDT suggests that an individual’s motivation to engage in a behavior, such as PA, is based on the satisfaction of their basic psychological needs of competence, autonomy, and relatedness [50]. From an SDT perspective, interventions can enhance needs satisfaction by providing autonomy support (ie, providing PA choices and minimizing perceived pressure) and structure (ie, communicating clear expectations, providing access to personalized information, and providing feedback) and by building strong interpersonal relationships with participants [50]. In turn, when an individual’s needs are satisfied and their motivation to engage in a behavior is internalized and for self-determined reasons, because PA is enjoyable, pleasurable, of interest, and aligned with their values, their behavior is more likely to become habitual and maintained over time. Both quantitative and qualitative studies support these tenets [29,53,54]. SCT suggests that self-efficacy is a key determinant of PA [51]. From an SCT perspective, interventions fostering an individual’s perceived self-efficacy via positive reinforcement can help promote PA uptake and adherence [55].

Using SDT and SCT when planning PA behavior change interventions, several studies have shown that targeting their underlying theoretical constructs (eg, perceptions of competence, autonomy and relatedness, motivational regulations, and self-efficacy) and introducing evidence-based motivational and behavior change techniques (MBCTs) can have a positive effect on PA outcomes [27,44,56]. MBCTs include shaping knowledge (ie, providing instruction on how to perform the behavior and how to monitor the behavior and affect), comparison of outcomes (ie, discussing pros and cons), natural consequences (ie, providing information about the health benefits of PA and monitoring of benefits or outcomes), goal setting (ie, setting goals, problem solving, action planning, discrepancy between current behavior and goal, review outcome goal or goals, behavioral contract, and commitment), fostering self-beliefs via verbal persuasion about the capability to overcome barriers, focus on past success, and self-talk, feedback, and monitoring (ie, providing feedback on behavior, self-monitoring of outcome or outcomes of behavior, and self-monitoring of behavior), associations (ie, picking prompts and cues), and rewards and threats (ie, building self-rewards) [27,44,56]. Collectively, studies have shown that a variety of SDT- and SCT-based interventions, including MBCTs, are promising for increasing PA in people with overweight or obesity [56,57]. However, no single theory has shown superiority in its ability to increase PA, and there is an increasing trend to include multiple MBCTs that map onto multiple theories to ensure that several key factors influencing PA are targeted [52,56]. As such, rather than choosing one theory, PA behavior change interventions including MBCTs related to SDT and SCT constructs should be considered, as they may increase the likelihood that patients awaiting MBS will initiate and maintain their PA.

### Approaches to Delivering Interventions

PA behavior change interventions for adults with obesity include center-, community-, and home-based programs. However, alternative delivery formats, including email, phone, or videoconferencing counseling, have also been shown to be effective [58]. Videoconferencing counseling, otherwise referred to as telehealth interventions, have been introduced to address key issues and patients’ preferences (eg, lack of time and resources, supervision, face-to-face, accessibility, and simplicity) while considering current contextual challenges (eg, limited financial and human resources, travel to and from MBS or research centers, pandemic lockdown, and environmental concerns) and maintaining opportunities for meetings with health professionals as per patient preferences. Therefore, it is important to develop and implement behavior change interventions using telehealth.

Telehealth refers to the use of the internet and connected technologies to provide care [59], and adherence rates as acceptability of such interventions are high (≥75%) [60-64]. The feasibility of telehealth interventions is very promising given the ubiquitous access to the internet and technologies in Canada, with 94% of residents having access to the internet and 88% owning a smartphone [65]. Moreover, telehealth is cost-effective and has already been shown to improve health outcomes [60,62,66] and PA among adults with or without obesity [67,68]. Although many studies are available on telehealth interventions with patients who had undergone MBS [69-74], to our knowledge, only 3 of them included a PA behavior change intervention: 1 feasibility study before MBS (before-after study design) [72]; 1 feasibility study after MBS (before-after study design) [73]; and 1 ongoing randomized controlled study after MBS [74]. However, PA was generally self-reported and was only measured in the short term, introducing measurement bias and preventing the assessment of PA maintenance [71]. Robust and innovative study designs, such as a single-case experimental design with long-term objective PA measures, should be used in future studies to assess the effects of telehealth PA behavior change interventions in the short and long term. Therefore, Bari-Activ, having already shown its efficacy [22,28,29], was revised to reinforce theory-based content, reflect intended users’ perspectives and the context in which the intervention will be conducted, and provide a means to increase reach (via telehealth). It will be tested within a single-case experimental design to ensure that it is feasible and applicable to intended users and implementers and generate data to inform a large-scale trial to test whether it is effective.

### Objectives

This manuscript reports the protocol used in this study. The first objective is to assess the feasibility and acceptability of the protocol or methods and the Telehealth Bariatric Behavioral Intervention (TELE-BariACTIV), an intervention designed to increase MVPA that is guided by two perspectives: (1) a multitheory approach, thus targeting SDT and SCT constructs and integrating related MBCTs, and (2) a patient perspective. The second objective is to generate an estimate of the effect of the TELE-BariACTIV intervention on presurgical MVPA (the primary outcome for a future large-scale trial) to determine the appropriate sample size for a multicenter large-scale trial.

## Methods

### Study Design and Procedures

The study uses a phased approach to behavior change intervention development supported by the Obesity Related Behavioral Intervention Trials model [31].

The study results will be reported according to the recommendations for single-case protocols in behavioral interventions [75]. Owing to the objectives of the study (feasibility, acceptability, and effect size exploration) and recommendations [31,76], this study is a multicenter study with a single-case experimental design with multiple base levels (ABAB’A). It is an open-label trial in which research staff, researchers, and participants are not blinded to the study objectives, intervention, and allocation, given the nature of the intervention and measures.

**Figure 1:**
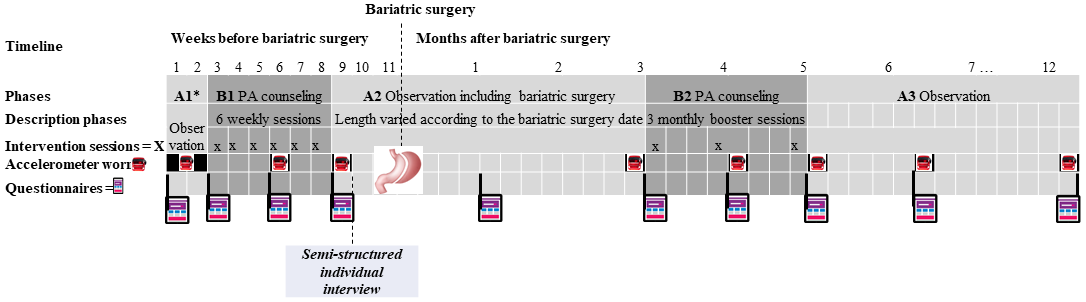
Study design.

Approximately 2 weeks before the baseline assessment, the study will be explained by phone to potential participants and the selection criteria, checked. Eligible and interested participants will be sent an accelerometer and a A370 Polar (Polar Electro) watch with cables to recharge and information sheet by mail with a prepaid return envelope. Participants will be instructed to initiate the baseline assessment on the web (Limesurvey) once they receive their packages, including an informed consent form. Then, participants will be randomly allocated to a 1- or 2-week baseline A1 phase using a list of random numbers generated electronically by a person outside the project, and they will start the 5 phases of the study (A1-B1-A2-B2-A3; Figure 1). The A phases are observational phases without intervention, and the B phases are intervention phases with PA counseling delivered via Zoom technology platform. Participants had different dates of starting the study owing to the variability in their MBS dates. Phase A2 may also vary in length because the exact MBS date is usually not known when participants are to start the study.

To meet the design standards that recommend at least 3 measurements per phase [75,77], MVPA is assessed continuously over 7 to 14 days during each phase. The storage limit of the accelerometer is 180 days, which ensures the feasibility of the measurement. Therefore, participants have to download the free Actisync application on a computer, connect the device with a cable, and then upload their anonymized accelerometry data using the Actigraph Centerpoint minimum every 180 days. Quality of life, anxiety and depressive symptoms, motivational regulations, basic psychological needs satisfaction and frustration, self-efficacy to overcome barriers to PA, PA enjoyment, and social support for exercise are assessed using a series of validated questionnaires 6 to 10 times via Limesurvey (Table 1). In addition, participants are asked to respond to questions regarding the acceptability of the protocol and intervention in web-based questionnaires before and after the intervention (phase B1), as well as during a web-based semistructured individual interview 1 to 4 weeks after phase B1. Medical records will be consulted at the end of the study with the participants’ authorization to validate the MBS date, medical conditions, and weight loss.

**Table 1:** Description of the assessment of the study outcomes, all completed in French.

Outcomes			Assessment methods and tools	Number of assessments and timing
**Primary outcomes**				
	Feasibility		Refusal, recruitment, retention, attendance, attrition, and missing data rates tracking by study staff	Throughout the study
	Acceptability		Technical Quality Assessment Questionnaire Semistructured individual interviews Acceptability questionnaire developed by the study authors	9; immediately after each intervention session during phases B1 and B2 1; 1 to 4 weeks after phase B1 2; 1 to 3 days before and after phase B1
**Secondary outcomes**				
	**Behavior targeted: MVPA^a^ (primary outcome for a future large-scale trial)**			
		MVPA (min/days)	Accelerometer (Actigraph)	8; during the 7 or 14 days of the phase A1, during 7 days before phase B2, mid- and after phases B1 and B2, as well as 6 and 12 months after MBS^b^ (phase A3)
	**Generalization measures (secondary outcomes for future large-scale trial)**			
		Health-related quality of life	RAND 36-item health survey	10; 1 to 3 days before phase A1, 1 to 3 days before, mid- and after phases B1 and B2, as well as 1, 6, and 12 months after MBS (phase A3)
		Anxiety and depressive symptoms	PHQ-9^c^ GAD-7^d^	10; 1 to 3 days before phase A1, 1 to 3 days before, mid- and after phases B1 and B2, as well as 1, 6, and 12 months after MBS (phase A3)
		Motivational regulations for PA^e^	BREQ2^f^	6; 1 to 3 days before and after each phase
		Basic psychological needs satisfaction and frustration	BPNSFS^g^	6; 1 to 3 days before and after each phase
		Self-efficacy for PA	Self-efficacity scale to overcome PA barriers developed by the study authors following Bandura’s recommendations	6; 1 to 3 days before and after each phase
		PA enjoyment	PACES^h^	6; 1 to 3 days before and after each phase
		Social support for exercise	SSES^i^	6; 1 to 3 days before and after each phase
	**Covariables**			
		Sociodemographic data	Questionnaire developed by the study authors	1; 1 to 3 days before phase A1
		Smoking status	Self-reported	6; 1 to 3 days before and after each phase
		Alcohol consumption	AUDIT-C^j^	6; 1 to 3 days before and after each phase
		BMI and weight loss	Self-reported medical records	6; 1 to 3 days before and after each phase
		Medications and medical conditions	Medical records	6; 1 to 3 days before and after each phase
		Date and place of MBS	Self-reported medical records	1; at the end of the study
		Pain	RAND 36-item health survey (bodily pain subscale)	10; 1 to 3 days before phase A1, 1 to 3 days before, mid- and after phases B1 and B2, as well as 1, 6, and 12 months after MBS (phase A3)
		Sedentary time, time spent on light-intensity PA, and step count	Accelerometer (Actigraph) GPAQ^k^	8; during the 7 or 14 days of the phase A1, during 7 days before phase B2, mid- and after phases B1 and B2, as well as 6 and 12 months after MBS (phase A3) 10; 1 to 3 days before phase A1, 1 to 3 days before, mid- and after phases B1 and B2, as well as 1, 6, and 12 months after MBS (phase A3)
		Self-reported sitting time and MVPA (min/week)	Accelerometer (Actigraph) GPAQ	8; during the 7 or 14 days of the phase A1, during 7 days before phase B2, mid- and after phases B1 and B2, as well as 6 and 12 months after MBS (phase A3) 10; 1 to 3 days before phase A1, 1 to 3 days before, mid- and after phases B1 and B2, as well as 1, 6, and 12 months after MBS (phase A3)

^a^MVPA: moderate-to-vigorous intensity physical activity.

^b^MBS: metabolic and bariatric surgery.

^c^PHQ-9: Patient Health Questionnaire.

^d^GAD-7: Generalized Anxiety Disorder scale.

^e^PA: physical activity.

^f^BREQ-2: Behavioral Regulation in Exercise Questionnaire-2.

^g^BPNSFS: Basic Psychological Need Satisfaction and Frustration Scale.

^h^PACES: Physical Activity Enjoyment Scale.

^i^SSES: Social Support for Exercise Survey.

^j^AUDIT-C: alcohol use disorder identification test–concise.

^k^GPAQ: Global Physical Activity Questionnaire.

### Participants

Participants are adults residing in or around 3 cities (ie, Montreal, Quebec City, and Chicoutimi) in the province of Quebec (Canada) and receiving care at either one of 2 tertiary care centers (Institut Universitaire de Cardiologie et de Pneumologie de Québec-Université Laval and the Centre Intégré Universitaire de Soins et de Services de Santé [CIUSSS] du Nord-de-l’Île-de-Montréal) or at a regional center (CIUSSS du Saguenay–Lac-Saint-Jean). Recruited strategies include (1) placing posters in waiting rooms at the target centers and (2) asking health professionals from hospitals at each site to refer to potentially eligible patients. For the latter, health professionals will provide the research team with the name and contact information of patients who have a planned sleeve gastrectomy in >12 weeks and who have provided verbal consent to be contacted by the research team for information on the study. The target sample size will be 12 (approximately 4 per site), which is considered acceptable for a single-case experimental study with multiple base levels, as participants serve as their own control, and outcomes will be measured repeatedly across observational and interventional phases [75,77].

The inclusion criteria are (1) ≥18 years of age; (2) scheduled to undergo sleeve gastrectomy in >12 weeks in one of the 3 associated hospitals (University Institute of Cardiology and Pulmonology of Quebec, Quebec; Sacré Cœur Hospital, Montreal [CIUSSS du Nord-de-l’Île-de-Montréal]; Chicoutimi Hospital, Chicoutimi [CIUSSS Saguenay–Lac-Saint-Jean]) Qc, Canada); (3) self-report ≤150 minutes of MVPA per week; and (4) access to a computer with internet and an interface with a camera. Exclusion criteria are (1) having a contraindication to PA without medical clearance (the Get Active questionnaire from the Canadian Society for Exercise Physiology [78] is used to determine if medical clearance is needed); (2) already enrolled in a supervised exercise intervention or PA behavior change intervention; (3) inability to speak and understand French; and (4) need a wheelchair, cane, walker, or other support or supports to move.

### Measurements

#### Primary Outcomes

##### Feasibility

The feasibility of the protocol or methods will be assessed at the end of the study using the following criteria tracked by the study staff: (1) refusal rate (percentage of participants who declined to participate); (2) recruitment rate (number of participants recruited per month at each center, number of sleeve gastrectomies performed per month at each center during the recruitment period), and sources of referral (eg, professionals, and self via poster); (3) retention rate (percentage of participants who complete all assessments and interviews); and (4) percentage of missing data (overall or total, per participant, per outcome, and per assessment time point). The feasibility of the intervention will be assessed at the end of the study using the following data tracked by the study staff: (5) attendance rate (number of sessions completed by participants) and (6) attrition rate (percentage of participants who did not complete the intervention). In addition, the reasons for refusing to participate in the study, as well as the reasons for dropping out of the study and the intervention, are documented by study staff, when available, and will be reported at the end of the study.

##### Acceptability

Immediately after each intervention session, the PA counselor completes the technical quality assessment questionnaire to report their satisfaction with the quality and performance of the Zoom technology platform [79]. The questionnaire includes 5 items: the first 3 concern the technological difficulties encountered by the PA counselor, and the last 2 relate to audio and video difficulties experienced by the participant. A high score indicates a low occurrence of technical problems. The questionnaire is modified for the study by adding 3 questions to assess the PA counselor’s perceived quality of the relationship with the participant, whether they believed the intervention session goals were met, and their overall satisfaction with the intervention session, with response options ranging from 0 to 10 (from lowest to highest satisfaction rate).

The acceptability of the protocol or methods and intervention is assessed during semistructured individual interviews with participants conducted via the Zoom platform. Participants are asked about their perceptions, opinions, and experiences regarding the intervention and study protocol or methods (eg, positive and negative experiences, adverse events related to the intervention and study, barriers, facilitators, satisfaction, perceived benefits, and suggestions for improvement). In addition, participants complete a 7-item questionnaire with statements in the future tense for before phase B1 or past tense for after phase B1, developed by the study authors (Arbour, G, unpublished data, July 2022), with items covering the 7 concepts of the *Theoretical Framework of Acceptability* by Sekhon et al [80] (ie, affective attitude, burden, ethicality, intervention coherence, opportunity costs, perceived effectiveness, and self-efficacy).

#### Secondary Outcomes

##### MVPA (Primary Outcome for the Future Large-scale Trial)

Daily MVPA is directly assessed using a triaxial accelerometer worn at the hip on the right side (Actigraph wGT3X-BT) during phase A1 and then for 7 days during the other assessment time points (Figure 1). Participants are instructed to wear the accelerometer continuously, except during water-based activities, and to maintain a log of wear times with waking and bed times. Accelerometer data will be extracted and downloaded (10-second epochs) using Actilife version 6.13.4 and will be cleaned in accordance with the logbooks provided by the participants. Only data from participants who wear the accelerometer for ≥3 days (including at least 1 weekday and 1 weekend day) and ≥10 hours/day will be analyzed [18,81]. The nonwear time is defined as a period of ≥120 minutes of consecutive zeros [82]. Accelerometer data will be used to quantify the time spent in MVPA (min/day). As PA intensity classifications have not yet been established for adults undergoing MBS, existing threshold values for the general population will be used for analyses (moderate-intensity PA=1952-5724 and vigorous-intensity PA 5724 counts/min) [83].

Data gathered immediately after phase B1 will be used to estimate effect sizes to determine sample size calculation for a future large-scale trial. Achieving PA recommendations of ≥150 min/week of MVPA will be judged as clinically significant [84,85].

##### Generalization Measures (Secondary Outcomes for the Future Large-scale Trial)

As recommended in the study by Tate et al [75], generalization measures are administered to increase the external validity of the study [86]. Generalization measures are dependent variables measured in addition to the targeted behavior to assess whether the effects of the intervention will be generalized to other results in the scientific literature [87]. In this study, generalization measures are used to assess changes in several psychosocial factors related to PA, spanning SDT, SCT, and health.

Health-related quality of life is assessed using the generic *RAND*-36 questionnaire [88], a 36-item questionnaire evaluating 8 concepts: physical functioning, role limitations owing to physical health, role limitations owing to emotional problems, energy or fatigue, emotional well-being, social functioning, pain, and general health perceptions. In addition, the *RAND*-36 contains an additional item to assess the perceived change in health status. The overall physical and mental health scores will be calculated from the 8 subscales [88]. A 3- to 5-point increase in overall physical and mental health scores is considered clinically significant [89,90]. According to the literature, owing to the likely absence of weight loss associated with the pre-MBS intervention, a score of 3 will be used as the minimal clinically important difference (MCID) [28,91].

The severity of anxiety and depressive symptoms will be assessed using the Patient Health Questionnaire (PHQ-9) [92] and the Generalized Anxiety Disorder scale (GAD-7) [93] as recommended by Obesity Canada [8]. The PHQ-9 is a 9-item validated scale that assesses the intensity of depressive symptoms. The GAD-7 is a 7-item self-rating scale that assesses the severity of anxiety symptoms. The MCID for the PHQ-9 and GAD-7 [94], based on the range of baseline depressive or anxiety symptom severity, will be used. Then, the MCID range will be 0 to 10 points (10/21, 48%) on the GAD–7 and 0 to 14 points (14/27, 52%) on the PHQ-9 for participants with baseline very mild to high anxiety or depression symptoms [94].

##### Theoretical Construct Measures (Secondary Outcomes for the Future Large-scale Trial)

To explore whether the intervention can change one or several of the theoretical constructs targeted, several measures are used to assess SDT, SCT, and evidence-based PA determinants [54,95,96], including PA enjoyment, motivational regulations for PA, psychological needs satisfaction and frustration, self-efficacy to overcome barriers to PA, and social support for exercise. PA enjoyment is assessed using the 18-item PA Enjoyment Scale [97]; motivational regulations for PA (ie, intrinsic, identified, introjected, integrated, external, and amotivation) are assessed using the 19-item Behavioral Regulation in Exercise Questionnaire-2 [95]; basic psychological needs satisfaction and frustration (ie, perceptions of autonomy, relatedness, and competence satisfaction and frustration) are assessed using the 24-item Basic Psychological Need Satisfaction and Frustration Scale [98,99]; self-efficacy to overcome common barriers to PA is assessed using a 20-item questionnaire developed by the research team according to Bandura’s recommendations. Participants are asked about their confidence to engage in PA 3 times per week in the presence of 20 different types of conditions using a 0% (“not confident at all”) to 100% (“highly confident”) scale [100], and perceived social support for PA received from family and friends is assessed using the 26-item Social Support for Exercise Scale [101,102]. Each measure will be scored following the scoring procedures, wherein higher scores will reflect greater PA enjoyment, intrinsic, identified, introjected, integrated, external, amotivation, self-efficacy to overcome common barriers to PA, psychological needs satisfaction, psychological needs frustration, and perceptions of social support for exercise.

##### Covariables

A questionnaire created by the authors is used to gather sociodemographic (age, sex, level of education, marital and professional status, number of children, and income) and clinical data (MBS date, medical conditions, and weight). The accuracy of the self-reported clinical data will be verified at the end of the study by reviewing the participants’ medical records. Smoking status (nonsmoker, smoker, or former smoker) is self-reported, and unhealthy alcohol consumption is assessed using the Alcohol Use Disorder Identification Test-Concise [103].

Sedentary time (hours/day), light PA (min/week), and daily step count is assessed using accelerometers with the following threshold values used for analyses: sedentary time ≤100 counts/min, light-intensity PA=100-1951 min/week [83]. The Global Physical Activity Questionnaire [104] is also completed by the participants to obtain self-reported PA data because it allows for the estimation of participants’ levels of PA during previous 7 days in 3 domains (work, travel to and from places, and recreational activities), which is not possible with accelerometers [104]. Total self-reported MVPA (min/week) will be calculated by summing the MVPA scores for transportation, leisure, and work. The question— “How much time do you usually spend sitting or reclining on a typical day?”—in the Global Physical Activity Questionnaire will be used to assess self-reported sedentary time.

Pain is assessed using the *RAND*-36 Physical Pain subscale, which consists of the following two validated items: (1) How much bodily pain have you had during the past 4 weeks? (2) During the past 4 weeks, how much did pain interfere with your normal work (including both work outside the home and housework)? An average score will be computed, whereby higher levels will reflect greater pain levels.

### The TELE-BariACTIV Intervention

During phase B1, participants will receive 6 real-time, face-to-face sessions with the same PA counselor (to the extent possible) over a 6-week period before MBS. The sessions will last approximately 45 minutes and will be conducted via videoconferencing using Zoom. The TELE-BariACTIV intervention is a multicomponent intervention derived from a previous intervention by Bond et al [22,28,29], which was developed based on multiple theories, including SDT and SCT, and was revised by the current research team. The intervention targets SDT and SCT constructs, focusing specifically on (1) providing autonomy support, structure, and interpersonal involvement; (2) increasing perceptions of autonomy (ie, perceived control over one’s actions by providing a rationale, structure, and emphasizing responsibility), competence (ie, perceived mastery of tasks and skills by providing support and encouragement, information feedback, and support barrier identification), and relatedness (ie, perceived belonging and connection to others by encouraging to seek social support); (3) increasing autonomous motivation for PA (ie, acting through self-endorsement and volition because the activity holds inherent interest or personal value); and (4) fostering perceived self-efficacy to overcome PA barriers and engage in PA. Accordingly, the intervention integrates several content- and relational-based techniques hypothesized to target the theoretical constructs. The techniques are selected on the basis of the theoretical construct or constructs they are proposed to target, wherein the research team drew on published literature [105-108] to determine which techniques target specific SDT and SCT constructs to guide the selection. The MBCTs used to guide the content of the intervention sessions during phase B1 are presented in Table 2.

In addition, the PA counselor uses motivational interviewing techniques [108] (ie, empathic or reflective listening, asking open-ended questions, prompting the participant to ask questions, prompting participants to offer solutions, seeking permission to provide information and advice, shifting focus, supporting change or persistence, and showing unconditional regard) that align with SDT and SCT for the relational component of the intervention to support the delivery of the content. The goal of the intervention is to address participants’ mindset about PA, as well as their motivation to engage in PA, with the goal of supporting PA uptake and maintenance. To this end, participants are encouraged to progressively increase their daily PA to achieve the current PA guidelines of 150 minutes per week of MVPA by the end of phase B1 (before MBS) and to maintain or increase PA levels during phases B2 (after MBS) and A3 (at the 1-year follow-up). Owing to the goal of helping participants increase and maintain PA after MBS, participants are offered 3 booster sessions to discuss progress or relapses and work on PA psychosocial and cognitive processes already addressed during phase B1. These sessions are one-on-one sessions, occur at 1-month intervals, and are conducted via videoconferencing using Zoom. They last 45 minutes, though they can be shorter as the topics are not pre-established; rather, the participants are free to choose the topics according to their needs. Length and topics discussed are recorded by the PA counselor.

**Table 2:** Overview of the telehealth bariatric behavior intervention^a^.

Session number and overview	Topics	Content- and relation-based techniques (motivation and behavior change techniques, and motivational interviewing techniques)
1. Welcome to the trial	Discuss importance of PA^b^ within context of bariatric surgery Identify health risks of a sedentary lifestyle and health benefits of an active lifestyle Evaluate perceived benefits and personal barriers related to PA adoption Establish baseline daily average PA minutes and steps Discuss PA target Provide PA monitoring logbook, pedometer, and instructions for recording daily bout-related walking exercise minutes and steps Provide information about the benefits and costs of action or inaction to participants	Review specific guidelines for PA participation Discuss benefits of PA and elicit participants’ reasons for increasing PA Examine cost and benefit of current PA behavior and changing behavior Acknowledge any internal conflict regarding PA adoption Offer clear rationale for PA adoption Self–re-evaluation (explore congruence between values, goals, and lifestyle) Assess motivational readiness *Shaping knowledge*: Instruction on how to perform the behavior, and how to monitor behavior and affect *Comparison of outcomes*: Credible source, pros and cons *Natural consequences*: Information about health consequences
2. Goal setting for behavior resolution	Introduce goal-setting principles, set goals targeting behaviors to increase PA Identify pleasant or unpleasant aspects of PA Differentiate extrinsic and intrinsic motives and rewards Identify ways to make PA more enjoyable	Review SMART^c^ goal approach Facilitate short- and long-term goal development Emphasize enjoyable aspects of PA *Goal setting*: Goal setting, problem solving, action planning, discrepancy between current behavior and goal, review outcome goal or goals, behavior contract, and commitment *Self-belief*: Verbal persuasion about capability, focus on past success, and self-talk *Natural consequences*: Information about health consequences, salience of consequences, monitoring of emotional consequences, and anticipated regret *Scheduled consequences*: Reward alternative behavior
3. Building a preoperative PA program	Differentiate lifestyle and structured PA Brainstorm ways to increase lifestyle PA Teach talk test to gauge PA intensity Discuss making PA a habit Record of PA behavior Record of outcomes related to PA Instruction to perform behavior Prompt practice	Review methods of self-monitoring Encourage the use of a self-monitoring technique to evaluate progress postintervention *Goal setting*: Goal setting, problem solving, action planning, discrepancy between current behavior and goal, review outcome goal or goals, behavior contract, and commitment *Feedback and monitoring*: Feedback on behavior, self-monitoring of outcome or outcomes of behavior, self-monitoring of behavior *Self-belief*: Verbal persuasion about capability, focus on past success, and self-talk *Reward and threat*: Self-reward *Associations*: Prompts or cues
4. Creating an active environment: Making physical and social cues work for you	Environmental restructuring Identify positive environmental cues to increase PA Provide information on where and when to perform PA Identify strategies to eliminate or avoid inactivity cues Plan social support or social change	Review main types of social support Encourage participants to examine social network Develop reasons and plans to include others in their lifestyle changes Have participants examine their current environment and determine methods for creating a PA-promoting environment *Goal setting*: Goal setting, problem solving, action planning, discrepancy between current behavior and goal, review outcome goal or goals, behavior contract, and commitment *Feedback and monitoring*: Feedback on behavior, self-monitoring of outcome or outcomes of behavior, and self-monitoring of behavior *Antecedents*: Restructuring the physical environment, restructuring the social environment, and body changes *Social support*: Social support (unspecified), social support (practical), and social support (emotional)
5. Resolving issues and planning	Problem-solving Action planning Barrier identification or problem solving Increasing self-efficacy	Elicit potential barriers that participants may experience Develop plans to overcome barriers *Goal setting*: Goal setting, problem solving, action planning, discrepancy between current behavior and goal, review outcome goal or goals, behavior contract, and commitment *Feedback and monitoring*: Feedback on behavior, self-monitoring of outcome outcomes of behavior, and self-monitoring of behavior *Self-belief*: Verbal persuasion about capability, focus on past success, self-talk *Repetition and substitution*: Behavior substitution
6. Putting it all together and establishing commitment or habit	Develop new contract to facilitate commitment to maintenance of PA change consisting of short-, medium-, and long-term goals Relapse prevention or coping planning	Behavior contracting or self-liberation Social support or helping relationships Self–re-evaluation Mastery experiences *Goal setting*: Goal setting, problem solving, action planning, discrepancy between current behavior and goal, review outcome goal or goals, behavior contract, and commitment *Self-belief*: Verbal persuasion about capability, focus on past success, and self-talk *Feedback and monitoring*: Feedback on behavior, self-monitoring of outcome or outcomes of behavior, and self-monitoring of behavior *Antecedents*: Restructuring the physical environment, restructuring the social environment, and body changes *Social support*: Social support (unspecified), social support (practical), and social support (emotional) *Repetition and substitution*: Habit formation *Associations*: Prompts or cues

^a^Behavior change technique groupings refer to the hierarchically clustered 93 techniques presented in the behavior change technique taxonomy (v1), are italicized, and specific techniques to be used follow the colons.

^b^PA: physical activity.

^c^SMART: specific, measurable, attainable, relevant, time-based.

In addition, participants are encouraged to use the provided PA monitor (A370 Polar watch) for the study duration to track their PA behavior (not for research data collection), as this has been shown to be an effective way to increase goal setting, self-monitoring, and overall PA adherence [109,110].

PA counselors are persons with at least a bachelor’s degree in kinesiology or a related field (eg, nursing care and physiotherapy). They receive training and supervision from AB and JB. As part of their training, they are required to participate in a 2-hour training course facilitated by JB, AB, and Jenson Price on how to deliver the intervention. In addition, they are observed performing the mock counseling encounters and take part in a bracketing interview with JP to reflect on their own background, perceptions, and interests and to explore how these could impact them in their role. PA counselors also receive an intervention manual created by JB, AB, and JP and reviewed by PB, AJR, and DB, which details the intervention. Specifically, the manual provides session plans and suggested activities, as well as relevant worksheets and documents that are shared with participants via mail and email. Finally, notes for each session with duration, topics discussed, accomplishment of session objectives, participants’ reactions to the content, next steps with the participants, and reflection as a PA counselor (eg, problems arising during the sessions, overall experience delivering the session, suggestion for intervention or personal improvements, and session deviations) are taken by the PA counselor using a standardized form and discussed with AB, if needed. This information will be used to track fidelity to the intervention protocol and explore avenues for intervention improvement in future large-scale trials.

### Internal Validity

During the individual interviews, the participants are asked about all life events or factors that occurred during the study period (eg, divorce, injuries, illness, lockdown, and new employment) that may have impacted the study results. This strategy allows for greater certainty in confirming whether any changes observed are related to the intervention [111].

### Statistical Analyses

Descriptive statistics will be computed for baseline sociodemographic, clinical, feasibility, and acceptability data derived from the numerical responses and close-ended questions.

All statistical analyses will be carried out using R and the following packages: scan, SCRT [112], metafor, ggplot2, and tydiverse. For the primary outcomes of a future large-scale trial, a comparison of daily MVPA based on accelerometer data between phases A1 and B1 will be carried out using a randomization test for a single-case experimental design [113] for each participant. Subsequently, individual effect sizes will be aggregated in a meta-analysis to obtain a group-based effect size. This will provide the necessary information for computing the required sample size for a future large-scale trial. A sensitivity analysis will also be performed to compare A1 and B1 using a Tau-*U* test [114]. Then, a second meta-analysis will be performed using the same approach. The global effect of the intervention on daily MVPA will be tested using another set of randomization tests comparing the ABABA phases. This test makes it possible to compare 2 short time series and to provide an effect size in case of a significant difference. This analysis will be performed on an individual scale.

Generalization measures (secondary outcomes for a future large-scale trial: health-related quality of life, anxiety, and depression symptoms) will be analyzed using the Reliable Change Index or cutoff score for descriptive purposes; the differences in individual scores across phases will be compared [115].

Theoretical construct measures (secondary outcomes for a future large-scale trial: PA enjoyment, motivational regulations for PA, basic psychological needs satisfaction and frustration, self-efficacy to overcome PA barriers, and social support for exercise) and self-reported MVPA will be analyzed using visual analysis. As recommended [116], stability, mean level change, and range of data by phase will be examined.

Content analysis of participants’ responses to open-ended questions during the interviews and of the PA counselors’ notes completed after the session will be conducted to identify themes and see if or what changes to the protocol or methods or intervention are warranted [117]. This will involve five steps, which will be followed by 2 research staff members: (1) familiarize themselves with the data by reading each transcribed interview several times before coding and looking for patterns, (2) code the transcripts and compile the initial codes, (3) put together similar codes to form potential themes and subthemes, (4) review the main themes and subthemes in relation to the data, and (5) define and name themes, select illustrative quotes from the interviews and produce a report.

### Ethics Approval

The study protocol was approved by the Institutional Review Boards at the CIUSSS du Saguenay–Lac-Saint-Jean (approval MP-25-2021-354; 2021-6-11), the Institut Universitaire de Cardiologie et de Pneumologie de Québec-Université Laval (approval MP-25-2021-354; 2021-8-2), and the CIUSSS du Nord-de-l’Île-de-Montréal (approval MEO-25-2022-2264; 2021-10-22).

## Results

The study began in September 2021, and as of July 2022, 12 participants have provided informed consent and been enrolled. Data collection is expected to end in fall 2023, depending on the MBS date. Analysis of pre-MBS data is planned for fall 2022, with the manuscript expected to be submitted in spring 2023. Analysis of post-MBS data is planned for fall 2023, with a second manuscript expected to be submitted in spring 2024.

The achievement of the following criteria based on the literature [22,28,29,63,72,73,118] will be considered satisfactory: (1) refusal rate ≤20%, (2) recruitment rate ≥1.8 participants recruited per month, (3) retention rate after phase B1 (before MBS) ≥80%, (4) percentage of missing data after phase B1 (before MBS) ≤10%, (5) intervention attendance rate ≥80%, and (6) intervention attrition rate ≤20%.

## Discussion

### Principal Findings

Increasing MVPA uptake and maintenance in adults undergoing MBS is a public health priority. This study will provide critical evidence pertaining to the feasibility and acceptability of the TELE-BariACTIV aimed at promoting MVPA before and after MBS while addressing several barriers to MVPA and intervention engagement previously identified, as well as the protocol or methods proposed to evaluate it. The data collected in the individual interviews will help optimize the intervention and protocol or methods for a larger study. In addition, an estimate of the effect of the TELE-BariACTIV intervention on MVPA will be useful for calculating the required sample size for future multicenter large-scale trials.

This paper describes the protocol and intervention used in the TELE-BariACTIV study. To our knowledge, only 2 studies investigating PA behavior change interventions among adults undergoing MBS have been published [72,73]. The TELE-BariACTIV study extends these previous works by prolonging the duration of the intervention (to cover both before and after MBS) and accordingly integrates longer follow-up assessments to assess initial changes in PA and maintenance of PA. Furthermore, the intervention and protocol or methods were designed to be as accessible and simple as possible (ie, distance-based), with no need for in-person assessments or intervention sessions while still using an objective PA measure.

A single-case experimental study with multiple base levels (ABAB’A) design, in which a small group of participants is used to test causal relationships between variables of interest, is often used in behavior research because it allows rigorous experimental manipulation of independent variables and repeated measures of dependent variables over time [119]. This design has been used with various clinical populations to study different behaviors, including PA [87,120,121] in patients who have undergone MBS [122]. In addition, observational phases improve internal validity by controlling for maturation and historical variables, thus performing a function similar to that of the control group without intervention [75].

### Limitations

Although the TELE-BariACTIV intervention was developed according to patients’ preferences, some patients will like other options (eg, in-person intervention and supervised exercise training), and some will be excluded because they have no internet access or interface with a camera and need supportive devices to move, leading to potential enrollment difficulties and selection bias. The feasibility of the intervention in the general MBS population will not be assessed. However, this could be the goal of another larger study. In addition, recruitment began during the COVID-19 pandemic period. The COVID-19 pandemic has led to an overload on the health care system, which presumably slowed recruitment. Thus, the recruitment rate that will be estimated after the study is likely to be conservative. MBCTs are grouped together, so there is no way to know which technique has more or less impact on MVPA change. Nevertheless, the main aim of this initial study is to explore the effect of the intervention to calculate the sample size for a future large-scale trial and not to isolate the most efficacious intervention components. Finally, several outcome measures used are not specific to patients who have undergone MBS and may not be sensitive enough to capture slight changes that may be clinically meaningful for this population. Therefore, participants are offered an opportunity to share any additional thoughts they have, which can relate to any change in the outcomes measured during the interview.

To conclude, the TELE-BariACTIV study is innovative in its approach and design [76]. Feasibility and acceptability evidence would support proceeding with a larger trial that is sufficiently powered to test its effectiveness. Moreover, the TELE-BariACTIV intervention has strong potential for sustainability in the current context (pandemic, environmental, and budgetary restrictions). Indeed, given the ubiquity of the internet, TELE-BariACTIV intervention could be implemented across Canada to support cost-effective efforts to promote PA in this growing, mostly inactive, segment of the population. In addition, it would offer adults access to supportive care and trained professionals regardless of where they live. Finally, use of distance-based means to collect data to encourage participation of adults near and far from urban centers and use of technology to deliver the intervention to address participants’ lack of time and unwillingness to travel for in-person sessions are strengths of this study.

## Abbreviations

CIUSSS: Centre Intégré Universitaire de Soins et de Services de Santé
GAD-7: Generalized Anxiety Disorder scale
MBCT: motivational and behavior change technique
MBS: metabolic and bariatric surgery
MCID: minimal clinically important difference
MVPA: moderate to vigorous intensity physical activity
PA: physical activity
PHQ-9: Patient Health Questionnaire
SCT: social cognitive theory
SDT: self-determination theory
TELE-BariACTIV: Telehealth Bariatric Behavioral Intervention

## Data Availability

Data generated or analyzed during the study will be included in the published article and its supplementary information files. Additional information will be available from the corresponding author upon request.
